# *β*‐alanine supplementation in adults with overweight and obesity: a randomized controlled feasibility trial

**DOI:** 10.1002/oby.24204

**Published:** 2025-01-12

**Authors:** Joseph J. Matthews, Jade V. Creighton, James Donaldson, Paul A. Swinton, Ioannis Kyrou, Srikanth Bellary, Iskandar Idris, Lívia Santos, Mark D. Turner, Craig L. Doig, Kirsty J. Elliott‐Sale, Craig Sale

**Affiliations:** ^1^ Department of Geriatrics, Donald W. Reynolds Institute on Aging, Center for Translational Research in Aging & Longevity University of Arkansas for Medical Sciences Little Rock Arkansas USA; ^2^ Sport, Health and Performance Research Centre, School of Science and Technology Nottingham Trent University Nottingham UK; ^3^ Centre for Systems Health and integrated Metabolic Research (SHiMR), School of Science and Technology Nottingham Trent University Nottingham UK; ^4^ Department of Cardiovascular Sciences University of Leicester Leicester UK; ^5^ NIHR Leicester Cardiovascular Biomedical Research Unit Glenfield Hospital Leicester UK; ^6^ School of Health Sciences Robert Gordon University Aberdeen UK; ^7^ WISDEM University Hospitals Coventry and Warwickshire NHS Trust Coventry UK; ^8^ Research Institute for Health & Wellbeing Coventry University Coventry UK; ^9^ College of Health, Psychology and Social Care University of Derby Derby UK; ^10^ Laboratory of Dietetics and Quality of Life, Department of Food Science and Human Nutrition Agricultural University of Athens Athens Greece; ^11^ Aston University and University Hospitals Birmingham Foundation Trust Birmingham UK; ^12^ Centre of Metabolism, Ageing & Physiology, NIHR Nottingham Biomedical Research Centre, School of Medicine University of Nottingham Nottingham UK; ^13^ Department of Sport and Exercise Sciences Manchester Metropolitan University Institute of Sport Manchester UK

## Abstract

**Objective:**

Overweight and obesity are characterized by excess adiposity and systemic, chronic, low‐grade inflammation, which is associated with several metabolic disorders. The aim of this study was to assess the feasibility and tolerability of β‐alanine supplementation and to explore the effects on cardiometabolic health and cardiovascular, hepatic, and renal function in adults with overweight and obesity.

**Methods:**

A total of 27 adults (44% female; mean [SD], age: 58 [10] years, BMI: 31.1 [2.9] kg/m^2^, hemoglobin A1c: 39.8 [4.3] mmol/mol) received β‐alanine (4.8 g/day) or a matched placebo for 3 months. Feasibility and tolerability outcomes included adherence, side effects, recruitment, attrition, and blinding, and exploratory outcomes included biochemical markers, blood pressures, and transthoracic echocardiography parameters. Data were analyzed using a Bayesian approach presented with 95% credible intervals (CrI).

**Results:**

β‐alanine was well tolerated and adhered to (adherence: placebo, 0.91 [95% CrI: 0.84–0.95]; β‐alanine, 0.92 [95% CrI: 0.85–0.95]), and side effects remained at or below baseline throughout. The probability that β‐alanine supplementation affected cardiometabolic, cardiovascular, or clinical biochemical outcomes was low.

**Conclusions:**

Sustained‐release β‐alanine supplementation is well tolerated and adhered to in adults with overweight and obesity. Future research should consider more advanced metabolic conditions, which may benefit from longer duration supplementation.

**Study Importance**

**What is already known?**

- Overweight and obesity are characterized by low‐grade systemic inflammation and increases in reactive carbonyl stress.
- Carnosine and β‐alanine show promise as therapeutic supplements to improve cardiometabolic health and cardiovascular function.

**What does this study add?**

- This is the largest cumulative β‐alanine dose supplemented in a clinical population.
- We show that sustained‐release β‐alanine is well tolerated and adhered to in adults with overweight and obesity.

**How might these results change the direction of research or the focus of clinical practice?**

- These results suggest that β‐alanine may not improve cardiometabolic health and cardiovascular function in adults with overweight and obesity.
- Alternative dietary therapies and more advanced metabolic conditions should be explored.

## INTRODUCTION

Overweight and obesity are major public health problems. Recent estimates show that 64% of people in the UK are living with overweight and obesity, which equates to ~43.3 million people [1]. These conditions are characterized by excess adiposity and systemic, low‐grade inflammation associated with a range of metabolic disorders, including dyslipidemia, hypertension, and hyperglycemia [2], with an increased risk of developing prediabetes, type 2 diabetes (T2D), and cardiovascular disease (CVD) [3]. Exercise and dietary interventions can help delay or prevent disease progression by reducing body weight, systemic inflammation, and chronic oxidative stress [4, 5], although long‐term adherence is difficult to sustain and effectiveness varies among individuals [6]. Therefore, it is important to develop novel interventions to improve cardiometabolic health and cardiovascular function in individuals with overweight and obesity.

Carnosine is a multifunctional dipeptide with emergent roles in health and disease [7]. It exists in high concentrations in human skeletal muscle (~22 mmol/kg/dry weight) and lower concentrations in human cardiac muscle (~26 μmol/kg/dry weight) [8]. Skeletal and cardiac muscle concentrations can be increased by up to twofold [9] and sevenfold [10] with prolonged supplementation of its rate‐limiting precursor, β‐alanine. Carnosine may improve cardiometabolic health through its role as a scavenger of reactive carbonyl species (RCS), where it forms stable adducts that can be metabolized and excreted from the body [11], and via Ca^2+^ handling in cardiac muscle, where it acts as a Ca^2+^‐H^+^ exchanger to improve excitation‐contraction coupling [12]. In support of this, a meta‐analysis showed that supplementation with carnosine or β‐alanine reduced fasting glucose and hemoglobin A1c (HbA1c) in humans and rodents beyond minimal important difference thresholds (≥1 mmol/L and ≥0.5% reductions in humans) [13]. Carnosine has been shown to decrease RCS‐modified proteins and increase insulin‐stimulated glucose uptake in T2D skeletal muscle cells and C2C12 myotubes cultured under glucolipotoxic conditions [14, 15]. These studies suggest a potential role for carnosine and β‐alanine in obesity, prediabetes, T2D, and CVD.

While initial randomized controlled trials (RCT) have shown positive effects of supplementation, these studies have supplemented participants with small doses of carnosine (0.5–2 g/day) for 12 weeks, equivalent to 0.20 to 0.79 g/day of β‐alanine (total intake: 16–67 g) [16, 17, 18, 19, 20, 21]. These intakes are likely to have a modest effect (~5%–20% increase) on skeletal muscle carnosine stores [22], whereas supplementing with high‐dose β‐alanine for the same duration can lead to greater increases and may potentiate its therapeutic effects. One concern is that high‐dose β‐alanine can cause paresthesia [23], although this may be mitigated with sustained‐released formulas that also have superior bioavailability [24]. Further limitations with existing trials include a lack of rigorous randomization, allocation concealment, and assessment of adherence, which could influence study outcomes. Therefore, the primary aim of the present study was to assess the feasibility and tolerability of β‐alanine supplementation in adults with overweight and obesity. A secondary aim was to explore the effect of supplementation on cardiometabolic health and on cardiovascular, hepatic, and renal function. Collectively, the study aimed to generate novel information on β‐alanine and carnosine to inform the design of future intervention studies.

## METHODS

### Trial design

The trial was a 3‐month, randomized, triple‐blinded, placebo‐controlled feasibility study, with parallel groups allocated 1:1. Study procedures received ethical approval from Nottingham Trent University and the Health Research Authority (REC reference: 21/NW/0280) and were conducted in accordance with the Declaration of Helsinki. All participants provided written informed consent. The trial was preregistered at ClinicalTrials.gov (NCT05329610). All protocol deviations from the preregistration are reported with explanations, and reporting follows the updated Consolidated Standards of Reporting Trials (CONSORT) guidelines [25].

### Participants

Participants were male and female individuals aged 18 to 75 years with a body mass index (BMI) ≥ 25 and <40 kg/m^2^ who were able to provide informed consent. Exclusion criteria were as follows: weight loss or gain ≥5 kg in the prior 6 months; participation in another research trial; substance abuse; mental health illness requiring active treatment; known eating disorder or cognitive impairment; inability to understand conversational English; diagnosed type 1 diabetes or T2D; use of carnosine or β‐alanine supplements in the prior 6 months; current breastfeeding, pregnancy, or consideration of pregnancy; use of weight‐loss or glucose‐lowering drugs (e.g., orlistat, thyroxine, metformin, glucagon‐like peptide‐1 analogues); long‐term corticosteroids; and known comorbidities that could impact study aims or outcomes (e.g., heart failure, chronic kidney disease, hemoglobinopathy).

Participants were recruited from primary care services via local general practice electronic database screening (termed: primary recruitment), and recruitment posters were circulated in local community groups, social media groups, libraries, the local newspaper, university campus, and the university website (termed: secondary recruitment). Owing to low response and enrollment rates, there was a change to the eligibility criteria after trial registration that removed the criteria for participants to have prediabetes based on HbA1c values recorded during visit one (42–47 mmol/mol) [26]. These changes were approved by the Health Research Authority, with further details on recruitment and enrollment reported in Table S1.

Table 1 depicts the trial procedures. Following initial telephone screening, participants attended laboratory screening and provided baseline measurements (visit one). Eligible participants returned 1 week later for further baseline measures and to begin the intervention (visit two), with the first dose taken under researcher supervision. Over the next 3 months, participants received two telephone calls before attending the final laboratory session (visit three), where all baseline measures were repeated. In accordance with the study design, the target enrollment was 30 participants to accommodate a 20% attrition rate (minimum of 12 participants per arm) [27]. Group allocation was performed by a statistician (who was not involved with recruitment, enrollment, or outcome assessment) via minimization, with sex, age, BMI, and HbA1c used as prognostic factors to minimize group imbalances [28]. All members of the research team were blinded throughout the study, and groups were not unmasked until all data were analyzed. Data collection and sample analyses were carried out in the Interdisciplinary Science and Technology Centre at Nottingham Trent University, Nottingham, UK.

**TABLE 1:** Overview of trial procedures.

Procedure	Visit 1	Visit 2	Visit 3
Health history questionnaire	●		
Height	●		
Body mass	●		●
Waist circumference	●		●
Handgrip strength	●		●
20‐mL blood sample	●		●
30‐mL urine sample	●		●
GASE questionnaire	●	●	●
CNAP measurements		●	●
TTE measurements		●	●
TDEE measurements	●		●
Collect and begin intervention		●	

Abbreviations: CNAP, continuous noninvasive arterial plethysmography; GASE, general assessment of side effects; TDEE, total daily energy expenditure; TTE, transthoracic echocardiography.

### Intervention

Participants received commercially available sustained‐release β‐alanine (Natural Alternatives International, Inc.) or a matched placebo containing microcrystalline cellulose (Natural Alternatives International) at a dose of 4.8 g/day (four doses of 2 × 600 mg tablets) for 3 months (432‐g total intake). This dose was chosen to increase skeletal muscle carnosine contents by ~60% to 80% [9, 29]. In order to promote adherence, participants were provided with a pillbox organizer and supplement diary and received a weekly automated text message throughout the study (FireText Communications Ltd.).

### Outcomes

Feasibility and tolerability outcomes were categorized as primary (adherence), secondary (recruitment, attrition, side effects, and blinding to the intervention), and exploratory (cardiometabolic outcomes; Table S2). Adherence was recorded at three time points (1 month, 2 months, and follow‐up) from supplement diaries and tablet counts (adherence = actual supplement ingested [grams]/expected supplement ingested [grams]). Recruitment of participants and attrition rates were recorded as proportions. For blinding to the intervention, participants were asked which intervention they thought that they had received, i.e., “β‐alanine,” “placebo,” and “don't know,” scored as +1, −1, and 0, respectively, with data reported descriptively. Side effects were recorded using a modified version of the General Assessment of Side Effects (GASE) questionnaire [30] at five time points (i.e., baseline, after the first supplementation dose, 1 month, 2 months, and follow‐up). The questionnaire was modified by adding a section on symptoms specific to paresthesia, which is a known side effect of β‐alanine supplementation [23]. Participants rated side effects across 47 domains (paresthesia: 10 domains, general: 37 domains) on a 0 to 3 scale representing “not present,” “mild,” “moderate,” and “severe,” respectively. Results were aggregated and reported descriptively as a median (range) value for side effects across each time point.

For visits one and three, participants attended the laboratory (~08:00–10:00 h) after an overnight fast; for visit two, participants were permitted to eat beforehand. For all visits, participants avoided caffeine for 12 hours prior and strenuous exercise and alcohol for 24 hours prior, which was confirmed verbally upon arrival. Participants consumed similar food and drink on the day before visits one and three, which was confirmed via 24‐h dietary recall. Height, body mass, and waist circumference were measured using standard protocols, and handgrip strength was measured using the Southampton protocol [31] with a hydraulic hand dynamometer (Jamar, Patterson Medical Supplies). Fasting blood samples were taken from the antecubital fossa using the venipuncture method with participants in a rested state. HbA1c was measured immediately in whole‐blood samples (Quo‐Test A1c, EKF Diagnostics), and remaining samples were centrifuged at 2000*g* for 10 min at 4°C and then stored at −80°C until analysis. Serum samples were allowed to clot at room temperature prior to centrifugation. Fasting urine samples were collected and centrifuged at 400*g* for 10 min at 4°C and then stored at −80°C until analysis. Enzyme‐linked immunosorbent assays (ELISA) were used to measure changes in serum insulin and C‐peptide (Mercodia AB), along with plasma methylglyoxal and 4‐hydroxynonenal (Abbexa Ltd.). Remaining blood and urine markers were analyzed in duplicate using a clinical chemistry analyzer (Pentra C400, Horiba ABX S.A.); coefficient of variation data are reported in Table S3. Homeostatic model assessment (HOMA) parameters were calculated from fasting glucose and C‐peptide to estimate β‐cell function (HOMA2–%B), insulin resistance (HOMA2‐IR), and insulin sensitivity (HOMA2–%S) using the Oxford computer method [32]. The quantitative insulin sensitivity check index (QUICKI) was used as an additional measure of insulin sensitivity: QUICKI = 1/(log[fasting insulin] + log[fasting glucose]) [33].

A continuous noninvasive arterial blood pressure monitor was used to measure beat‐to‐beat blood pressure wave form and hemodynamic outcomes (CNAP, CNSystems Medizintechnik GmbH). Participants laid supine in a quiet, darkened room for a 20‐min continuous measurement. Brachial blood pressure calibration was performed at 0 and 10 min, following which 5 min of continuous data were collected and used for statistical analyses. Resting transthoracic echocardiographic (TTE) measurements were recorded using a portable ultrasound system and a 4‐MHz cardiac transducer, with images obtained and analyzed offline according to recommended guidelines [34], using commercially available software (EchoPAC, version 204.x, GE Medical Systems). Measurements were recorded in triplicate, with mean values calculated and reported. Stroke volume index and cardiac index were calculated by normalizing stroke volume and cardiac output ($\dot{Q}$) to body surface area using the Mosteller formula [35]. At the end of visits two and three, participants were fitted with an Actiheart 5 device (CamNtech Ltd.) to wear continuously for 22 h. From this, physical activity and heart rate data were used to estimate total daily energy expenditure (Actiheart 5 software version 5.1.24, CamNtech Ltd.). Participants were excluded from TTE analysis if their scans showed poor echogenicity and from Actiheart analysis if their data had to be auto‐filled, if they removed the device for sleeping, or if they removed the device due to a skin reaction from the electrodes.

### Statistical analysis

Feasibility and tolerability outcomes were assessed as proportions and natural frequencies, and inferential analyses were conducted within a Bayesian framework [36]. For recruitment, conjugate β‐binomial models using the flat prior β(1,1) were used to obtain posterior predictive distributions to estimate the number of individuals to assess in order to achieve *n* participants for randomization and follow‐up in any future study (with 80% and 90% predictive probability) [37]. Adherence was modeled using predictive distributions obtained from β‐regression with a group fixed effect and default weakly informative priors (*t*‐distribution with three degrees of freedom). Bayesian ANCOVA models using baseline value as a covariate were used to obtain posterior probabilities for exploratory outcomes. Informative priors were used for HbA1c, fasting glucose, fasting insulin, HOMA2‐%B, and HOMA2‐IR, based on effect size values presented in our previous meta‐analysis [13]. Sensitivity analyses were also conducted for these outcomes using default weakly informative priors (*t*‐distribution with three degrees of freedom), with the same approach used for all remaining outcomes. Inferences were made using posterior distributions of the group difference effect based on the median (0.5 quantile), 95% credible intervals (CrI), and the associated probability (*p*[>0]), where *p* indicates the proportion of the posterior distribution that is beyond zero (i.e., *p* = 0.5 means the posterior distribution contains an equal proportion greater than and less than zero). Analyses were conducted using the R wrapper package *brms* interfaced with Stan to perform Bayesian sampling [38]. Descriptive statistics are presented as mean (SD), unless otherwise stated, and inferential data derived from the ANCOVA model are presented as median (95% CrI).

## RESULTS

**FIGURE 1:**
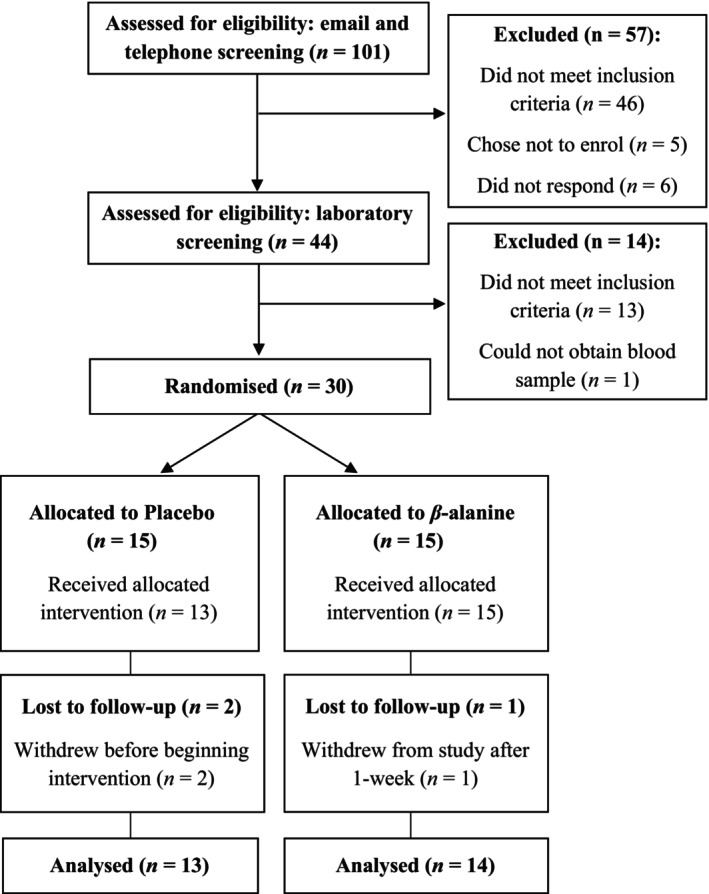
Consolidated Standards of Reporting Trials (CONSORT) flow diagram of study recruitment and attrition.

**FIGURE 2:**
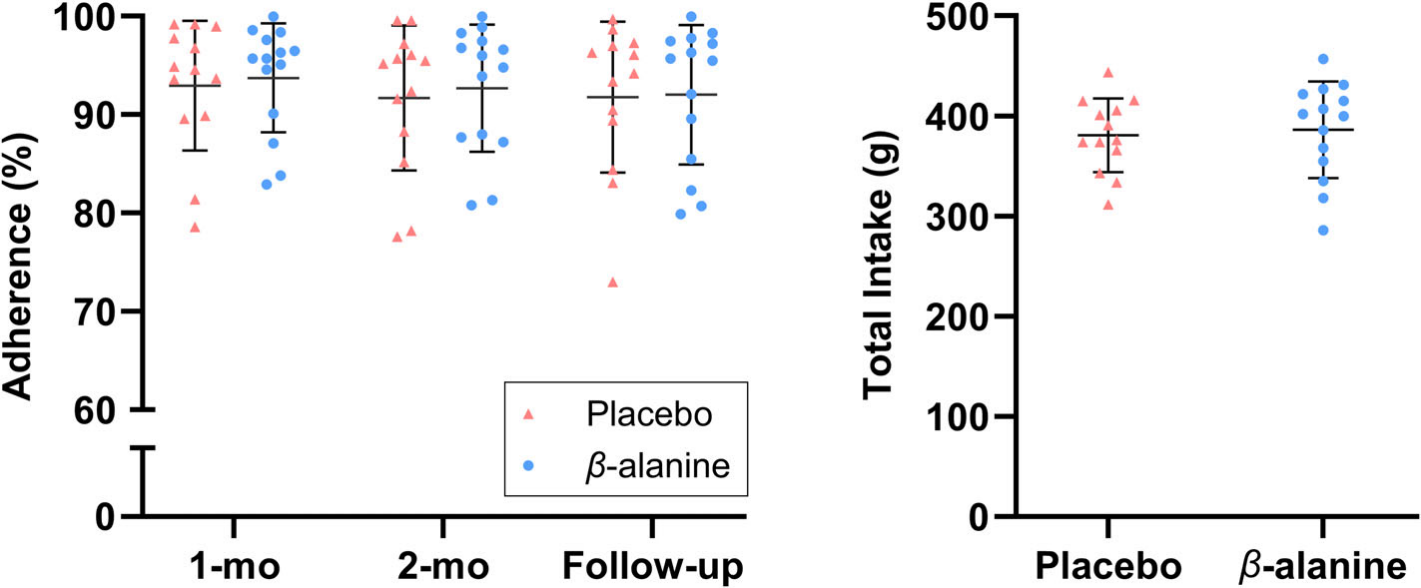
Supplement adherence across three time points; expressed as overall rate (percentage). Total supplement intake (grams) calculated from supplement adherence and duration in the study. [Color figure can be viewed at wileyonlinelibrary.com]

A total of 27 participants completed the trial (Table 2). Recruitment and follow‐up took place between March 2022 and July 2023. Figure 1 depicts the proportion of participants who attended laboratory screening (*n* = 44/101; 0.436), were randomized (*n* = 30/44; 0.682), and were evaluated for follow‐up (*n* = 27/30; 0.9). Point estimates of attrition were lower in the β‐alanine group compared with placebo (attrition rate: 6.7% and 13.3%); the two dropouts from placebo were prior to receiving the intervention, and the one dropout from β‐alanine was within 1 week of receiving the intervention (reason unrelated to the supplement). Adherence rates were similar across groups for observed values (at follow‐up: placebo, 0.91 [95% CrI: 0.84–0.95]; β‐alanine, 0.92 [95% CrI: 0.85–0.95]; Figure 2; Table S4). Paraesthesia side effects remained at or below baseline throughout (Table 3). The predictive distributions to obtain participants from *m* approached individuals for randomization and follow‐up were β‐binomial(*m*,31,72) and β‐binomial(*m*,28,75), which corresponded to *n* = 435 and *n* = 476 people screened to achieve *n* = 100 participants at follow‐up in a future trial (with 80% and 90% probability of achieving these targets); further distributions are reported in Table S5.

**TABLE 2:** Baseline participant characteristics.

	Placebo	β‐alanine
Number	13	14
Age, y	59 ± 10	57 ± 11
Sex, M/F *(n)*	8/5	7/7
Vegetarian (*n)*	1/13	1/14
Height, m	1.69 ± 0.13	1.70 ± 0.08
Body weight, kg	90.4 ± 15.3	89.2 ± 14.9
BMI, kg/m^2^	31.6 ± 3.0	30.6 ± 2.9
Waist circumference, m	1.03 ± 0.14	1.03 ± 0.13
Handgrip strength, kgf	37 ± 15	39 ± 15
TDEE, kcal/d	2585 ± 578	2690 ± 667
HbA1c, mmol/mol	40.2 ± 4.6	39.5 ± 4.0
HbA1c, %	5.8 ± 0.4	5.8 ± 0.4
Prediabetic‐NICE (*n*)	4	5
Prediabetic‐ADA (*n*)	9	9
Receiving antihypertensive or lipid‐lowering medications (*n)*	9	6

Abbreviations: ADA, American Diabetes Association prediabetes thresholds (≥5.7% to <6.5%); HbA1c, hemoglobin A1c; kgf, kilogram‐force; M/F, male/female; NICE, National Institute of Health and Care Excellence prediabetes thresholds (42–47 mmol/mol); TDEE, total daily energy expenditure.

**TABLE 3:** General assessment of side effects.

Time point	Placebo	β‐alanine
Paresthesia		
Baseline	0 (0–1) [8%]	0 (0–4) [43%]
Post‐supplementation	0 (0) [0%]	0 (0–1) [7%]
1 mo	0 (0–1) [8%]	0 (0) [0%]
2 mo	0 (0) [0%]	0 (0–4) [21%]
Follow‐up	0 (0–2) [8%]	0 (0–3) [7%]
General side effects^a^		
Baseline	4 (0–11)	5 (0–16)
Post‐supplementation	0 (0–6)	2 (0–10)
1 mo	2 (0–13)	2 (0–11)
2 mo	3 (0–19)	5 (0–12)
Follow‐up	3 (0–21)	3 (0–10)

*Note*: Baseline measures were recorded prior to the start of supplementation. Data presented as median aggregated score (range) and prevalence of paresthesia [%].

^a^ GASE questionnaire [30].

**FIGURE 3:**
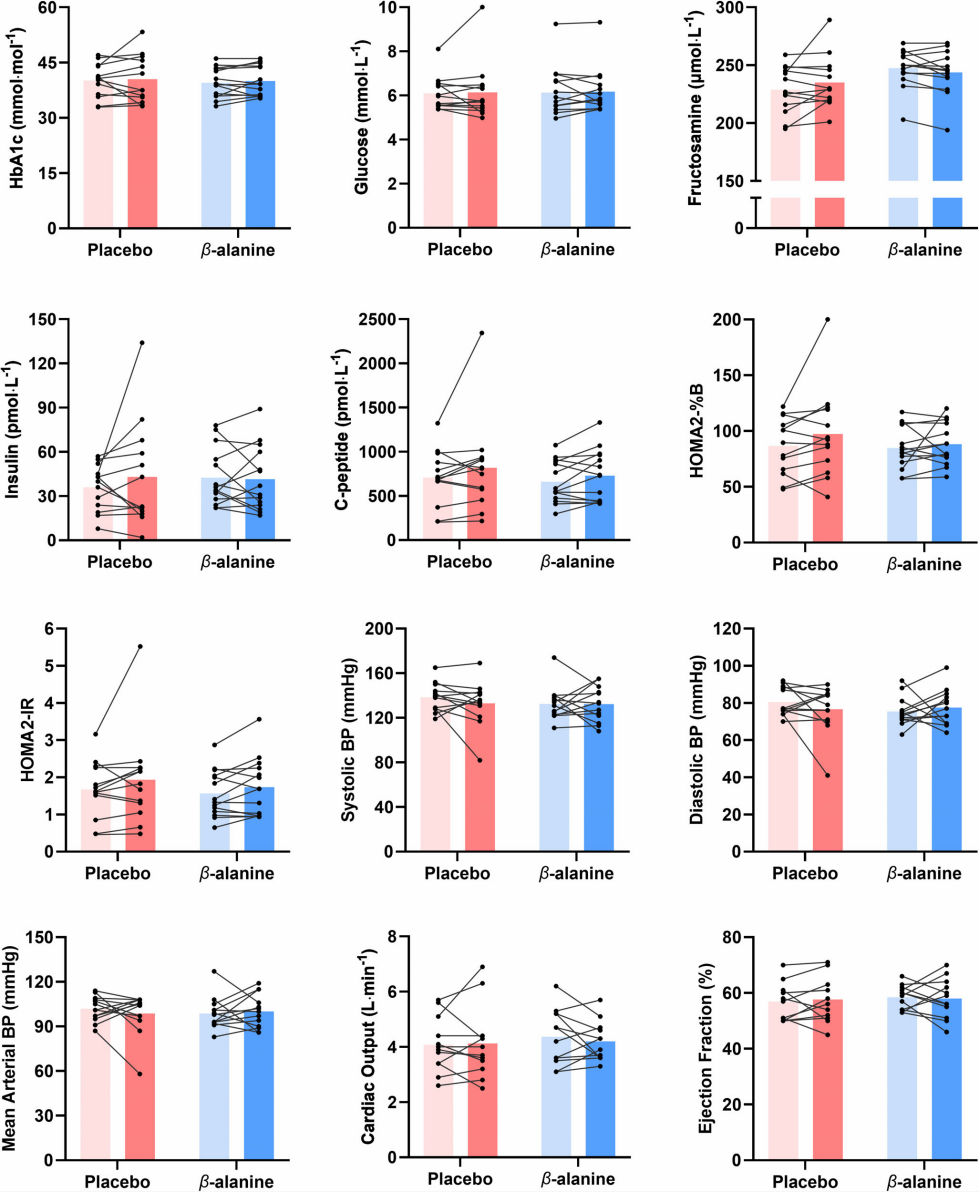
Pre‐ and post‐alanine supplementation values for selected cardiometabolic health and cardiovascular function outcomes. Data presented as mean values with individual data points. Statistical model inferential data are presented in Tables 4 and 5. [Color figure can be viewed at wileyonlinelibrary.com]

For outcomes with informative priors, results showed that β‐alanine supplementation may reduce fasting insulin, although there was large variability in this outcome (Table 4). The probability that β‐alanine supplementation affected other exploratory outcomes was low (Figure 3; Table 5; Tables S6 and S7). The strongest evidence was shown for β‐alanine reducing plasma fructosamine, although results for both groups remained within normal reference values. Urinary albumin data for one participant in each group showed high baseline and follow‐up values (β‐alanine, 222 and 119.3 mg/L; placebo, 491.9 and 1907.7 mg/L), which impacted the urinary albumin:creatinine ratio. A sensitivity analysis was performed without these participants (Table S6). Some preregistered outcomes were not explored due to the lack of changes in other relevant markers (Table S2). For blood pressures, the β‐alanine group comprised *n* = 13 due to missing data for one participant; for total daily energy expenditure, both groups comprised *n* = 11 due to measurement issues; and for TTE parameters (cardiac structure and function), the β‐alanine group comprised *n* = 12 for all outcomes, and the placebo group comprised *n* = 10 for structural outcomes and *n* = 12 for functional outcomes due to issues with echogenicity and image quality. Individual participant data are available open access: zenodo.org/records/14165012.

**TABLE 4:** Cardiometabolic outcomes with informative priors.

	Placebo		β‐alanine		Intervention effect			
Outcome	Baseline	Follow‐up	Baseline	Follow‐up	Default priors, placebo: β‐alanine	Prior used	Informative priors, placebo: β‐alanine	Probability > 0
HbA1c (mmol/mol)	40.2 ± 4.6	40.5 ± 6.4	39.5 ± 4.0	40.0 ± 4.1	0.14 (−2.4 to 2.6)	Normal (−10, 4^2^)	−0.79 (−3.3 to 1.6)	0.263
HbA1c (%)	5.8 ± 0.4	5.9 ± 0.6	5.8 ± 0.4	5.8 ± 0.4	0.02 (−0.21 to 0.25)	Normal (−0.91, 0.37^2^)	−0.06 (−0.30 to 0.15)	0.274
Glucose (mmol/L)	6.1 ± 0.8	6.1 ± 1.3	6.1 ± 1.1	6.2 ± 1.0	0.01 (−0.46 to 0.48)	Normal (−0.95,1.18^2^)	−0.02 (−0.50 to 0.45)	0.458
Insulin (pmol/L)	35.9 ± 15.6	43 ± 36.1	42.5 ± 19.4	41.4 ± 22.0	−8.5 (−26.9 to 9.9)	Normal (−7.2, 4.5^2^)	−7.5 (−15.2 to 0.47)	0.032
HOMA2‐%B	86.7 ± 25.5	97.3 ± 39.9	84.8 ± 19.1	88.2 ± 18.7	−7.2 (−23.9 to 9.7)	Normal (−4.8, 5.3^2^)	−5.5 (−14.3 to 3.5)	0.115
HOMA2‐IR	1.7 ± 0.8	1.9 ± 1.2	1.6 ± 0.6	1.7 ± 0.8	−0.07 (−0.50 to 0.37)	Normal (−0.28, 0.20^2^)	−0.18 (−0.48 to 0.11)	0.112

*Note*: Outcome data presented as mean ± SD; Bayesian ANCOVA inferential data presented as median (95% credible interval); the probability shows the proportion of the posterior distribution that is beyond zero (i.e., *p* = 0.5 means the posterior distribution contains an equal proportion greater than and less than zero). Abbreviations: HbA1c, hemoglobin A1c; HOMA, homeostatic model assessment; HOMA2‐%B, HOMA of β‐cell function; HOMA2‐IR, HOMA of insulin resistance.

**TABLE 5:** Cardiometabolic outcomes with default weakly informative priors.

	Placebo		β‐alanine		Intervention effect	Probability > 0
	Baseline	Follow‐up	Baseline	Follow‐up	Placebo: β‐alanine	
Body weight, kg	90.4 ± 15.3	90.8 ± 15.8	89.2 ± 14.9	89.2 ± 14.4	−0.44 (−2.2 to 1.3)	0.313
BMI, kg/m^2^	31.6 ± 3.0	31.7 ± 3.2	30.6 ± 2.9	30.6 ± 2.9	−0.12 (−0.72 to 0.49)	0.346
Waist circumference, m	1.03 ± 0.14	1.03 ± 0.13	1.03 ± 0.13	1.02 ± 0.12	−0.01 (−0.04 to 0.01)	0.143
Handgrip strength, kgf	37 ± 15	35 ± 13	39 ± 15	38 ± 16	0.86 (−2.3 to 4.0)	0.708
TDEE, kcal/d	2585 ± 578	2649 ± 521	2690 ± 667	2811 ± 824	84.8 (−373.9 to 543.6)	0.644
Systolic BP, mm Hg	138 ± 13	133 ± 20	132 ± 15	132 ± 16	2.2 (−11.4 to 15.9)	0.629
Diastolic BP, mm Hg	80 ± 8	77 ± 13	75 ± 8	77 ± 10	2.7 (−6.8 to 12.2)	0.721
MAP, mm Hg	99 ± 11	100 ± 11	102 ± 8	99 ± 14	2.8 (−7.1 to 12.8)	0.717
Cardiac output, L/min	4.1 ± 1.0	4.1 ± 1.3	4.4 ± 1.0	4.2 ± 0.7	−0.28 (−0.98 to 0.42)	0.207
Ejection fraction, %	57 ± 7	58 ± 8	59 ± 4	58 ± 7	−0.98 (−6.3 to 4.5)	0.355
C‐peptide, pmol/L	709 ± 316	819 ± 523	663 ± 238	730 ± 294	−30.1 (−211.7 to 151.3)	0.370
Fructosamine, μmol/L	229 ± 20	235 ± 22	247 ± 16	244 ± 19	−8.4 (−19.4 to 2.5)	0.067
hs‐CRP, mg/L	2.3 ± 1.9	1.9 ± 1.1	4.5 ± 6.0	3.4 ± 3.5	1.2 (−0.85 to 3.3)	0.886
HOMA2‐%S	82.3 ± 61.3	73.8 ± 51.9	74.6 ± 32.2	68.8 ± 28.7	1.1 (−11.3 to 13.6)	0.572
QUICKI	0.366 ± 0.041	0.382 ± 0.097	0.353 ± 0.028	0.357 ± 0.033	−0.00 (−0.04 to 0.03)	0.402
TC, mmol/L	5.0 ± 1.5	5.0 ± 1.6	5.3 ± 1.0	5.1 ± 1.2	−0.20 (−0.77 to 0.39)	0.242
LDL cholesterol, mmol/L	3.0 ± 1.1	3.0 ± 1.2	3.1 ± 0.9	3.0 ± 1.1	−0.09 (−0.54 to 0.38)	0.341
HDL cholesterol, mmol/L	1.3 ± 0.4	1.3 ± 0.4	1.3 ± 0.3	1.3 ± 0.3	−0.01 (−0.09 to 0.07)	0.408
Triglycerides, mmol/L	1.4 ± 0.5	1.4 ± 0.5	1.3 ± 0.4	1.3 ± 0.6	−0.05 (−0.31 to 0.23)	0.361
TC:HDL cholesterol ratio	4.1 ± 1.0	4.1 ± 1.1	4.1 ± 0.9	4.0 ± 1.2	−0.04 (−0.44 to 0.36)	0.416
LDL:HDL cholesterol ratio	2.5 ± 0.8	2.4 ± 0.9	2.4 ± 0.8	2.4 ± 1.0	0.03 (−0.30 to 0.36)	0.565
4‐hydroxynonenal	4.4 ± 2.4	2.7 ± 1.8	2.5 ± 1.4	2.5 ± 2.5	1.0 (−0.58 to 2.6)	0.901
MGO	11.7 ± 3.5	13.6 ± 2.7	13.4 ± 3.1	15.5 ± 3.8	1.8 (−1.0 to 4.6)	0.902

*Note*: Outcome data presented as mean ± SD; Bayesian ANCOVA inferential data presented as median (95% credible interval); the probability shows the proportion of the posterior distribution that is beyond zero (i.e., *p* = 0.5 means the posterior distribution contains an equal proportion greater than and less than zero).

Abbreviations: BP, blood pressure; HOMA2‐%S, homeostatic model assessment of insulin sensitivity; HDL, high‐density lipoprotein; hs‐CRP, high‐sensitivity C‐reactive protein; kgf, kilogram‐force; LDL, low‐density lipoprotein; MAP, mean arterial pressure; MGO, methylglyoxal; QUICKI, quantitative insulin sensitivity check index; TC, total cholesterol; TDEE; total daily energy expenditure.

## DISCUSSION

In this feasibility study, we showed that high‐dose sustained‐release β‐alanine was well tolerated and adhered to over a 3‐month period. This was evidenced by high adherence rates, low attrition after receiving the intervention, and participant‐reported side effects that remained at or below baseline values. This is the first RCT to quantify longitudinal adherence rates and evaluate the side effects for β‐alanine supplementation in a clinical population. Our results showed that, despite the high supplement burden (four doses of two tablets per day), the intervention was well adhered to throughout the trial, with higher adherence rates than similar trials using nutritional supplements such as vitamin D or omega‐3 tablets [39, 40]. These results provide evidence in favor of the strategies used to promote adherence—weekly automated text messages and monthly telephone calls—both of which have been shown to improve medication compliance across a range of populations [41, 42], in addition to tablet containers and hard‐copy supplement diaries. Although changes in skeletal muscle carnosine contents were not measured in this study, the total cumulative β‐alanine intake (mean [SD] 386 [48] g) was sufficient to increase skeletal muscle carnosine contents by ~60% based on previous findings [9, 29]. This was important to establish as it could influence the RCS‐scavenging capacity and therapeutic effects of muscle carnosine.

Participant‐reported side effects showed a lower prevalence of paresthesia than previous investigations [23], potentially due to the smaller individual dose and the slow‐release β‐alanine formula, which both result in lower peak plasma concentrations. A consequence of paresthesia is that it can lead to unintentional unblinding, whereby participants experience a known side effect and become aware of their group allocation. It was therefore important to establish successful blinding to β‐alanine supplementation, which provides a basis for future study designs. In the present study, markers of renal and hepatic function were within normal reference values at baseline and following β‐alanine supplementation. One study in children with type 1 diabetes and moderate albuminuria at baseline showed a large decrease (−58%) in the urinary albumin:creatinine ratio with carnosine supplementation, suggesting that carnosine might have a protective effect on kidney function (and lower the risk of diabetic nephropathy) [17]. However, other studies in adults with diabetic nephropathy have not shown a benefit with carnosine supplementation [21]. All other clinical outcomes were consistent with research in healthy athletes that shows β‐alanine is safe regarding biomarkers of renal and hepatic function [23, 43].

For some cardiometabolic outcomes, the analysis used informative priors from our previous meta‐analysis [13], which showed that β‐alanine supplementation may reduce serum insulin, although there was large variability in this outcome. These results are broadly in agreement with a recent human RCT that supplemented carnosine (2 g/day for 14 weeks) in adults with prediabetes and T2D and showed no effect on fasting glucose, HbA1c, fasting insulin, or any HOMA parameters [18]. Interestingly, the previous study showed a small effect of supplementation on 90‐ and 120‐min glucose and total area under the curve following an oral glucose tolerance test. However, this finding should be viewed alongside the lack of change in HbA1c, which would be expected to decrease alongside an improvement in glucose tolerance [44]. Our results are consistent with previous research on blood pressures and echocardiographic outcomes [20, 45]. It is worth noting that previous studies have shown small decreases in blood lipids, including total cholesterol [17] and fasting triglycerides [17, 19], but this is not consistent across the literature [16, 45]. These data, in conjunction with the present results, suggest that supplementation for durations of up to 3 months may not affect markers of cardiometabolic health and cardiovascular function in adults with overweight and obesity.

The present study has several strengths that improve upon previous research, specifically, clear randomization and allocation concealment methods, assessment of adherence and blinding, unbiased statistical analysis, and groups matched for prognostic factors at baseline. This reduces the possibility of chance bias, particularly from studies with baseline imbalances that were not adjusted for in statistical analyses [16, 21]. The present study also has limitations, including the use of static measures of glycemic control and insulin sensitivity (HOMA and QUICKI), where dynamic measurements would provide more insight (e.g., oral glucose tolerance test, hyperinsulinemic clamp techniques). Cardiometabolic and cardiovascular outcomes should be interpreted in line with the sample size and feasibility study design. In order to further explore effects on cardiometabolic health, a longer supplementation period may be beneficial, incorporating serial measurements over time. Several participants were taking prescribed antihypertensive and lipid‐lowering medications, which may negate the effect of supplementation on cardiovascular‐related outcomes. It remains unclear whether β‐alanine may be effective in populations with more severe metabolic conditions (e.g., T2D) or CVD.

## CONCLUSION

High‐dose sustained‐release β‐alanine supplementation for 3 months is well tolerated and adhered to in adults with overweight and obesity The probability that β‐alanine supplementation affected cardiometabolic health or cardiovascular, hepatic, and renal function was low. Consequently, our estimates suggest that a fully powered RCT to detect potential small effects in the present population would require a substantial sample size, and the predictive distributions provide estimates for recruitment numbers. Future research should consider alternative dietary approaches or more advanced metabolic conditions, which may benefit from supplementation.

## FUNDING INFORMATION

Joseph J. Matthews was funded by Birmingham City University. Jade V. Creighton was on a match‐funded studentship between Nottingham Trent University and Natural Alternatives International (NAI), a company formulating and manufacturing customized nutritional supplements. Craig L. Doig received Quality Research funding for this study from Nottingham Trent University.

## CONFLICT OF INTEREST DISCLOSURE

Natural Alternatives International (NAI) has provided Craig Sale with supplements for other studies free of charge and has contributed to the payment of open‐access publication charges for some manuscripts on β‐alanine supplementation. Craig Sale has also received an honorarium from NAI to produce materials to support a blog on β‐alanine supplementation and the effects of carnosine. NAI provided the supplements used in the trial but had no role in the design, methods, or analysis of the trial. The other authors declared no conflicts of interest.

## CLINICAL TRIAL REGISTRATION

ClinicalTrials.gov identifier NCT05329610.

## Supporting information


**Data S1:** Supporting Information.

## Data Availability

Deidentified individual participant data that underlie the results reported in this article are available in an open‐access repository (zenodo.org/records/14165012).
