# Supplementary material for: β‐alanine supplementation in adults with overweight and obesity: a randomized controlled feasibility trial
Source: Obesity (Silver Spring). 2025 Jan 12;33(2):278–88. doi: 10.1002/oby.24204 (PMC11774002; doi:10.1002/oby.24204)
Supplement: Supplementary file 1 — Data S1: Supporting Information. [file OBY-33-278-s001.docx]

| **Table S1.** Additional study recruitment and enrolment information. | |
| --- | --- |
| Local General Practice’s (GPs) screened their electronic databases for adults that met the following criteria: aged 18 to 75 years, BMI ≥25 and <40 kg^.^m^2^, and HbA1c of 42 to 47 mmol/mol. Letters containing the participant information sheet were sent out to eligible individuals (termed: primary recruitment). Recruitment posters were circulated in local community groups, social media groups, the University campus and website, and the local newspaper (termed: secondary recruitment). Prospective participants were required to contact the Research Team to arrange a telephone screening appointment.  Due to a low response and enrolment rate, an amendment was filed with the Health Research Authority to remove the requirement for HbA1c of 42 to 47 mmol/mol at baseline. Participants who had initially attended laboratory screening but were ineligible due to a HbA1c <42 mmol/mol, were contacted via email and re-invited to the study. In this second phase, primary recruitment involved four GPs (two new GPs and two in which participants were re-contacted). Recruitment posters were updated and recirculated in the same locations.  The first recruitment phase lasted five months (Mar to Sept 2022), during which five GPs distributed 839 recruitment letters. From this, 41 individuals contacted the Research Team (letter response rate: 4.9%) and four participants enrolled in the trial (letter enrolment rate: 0.48%), which equalled one participant enrolled per 210 letters distributed. We screened six individuals from secondary recruitment strategies, of which two enrolled in the trial. This low response and enrolment rate prompted the change in eligibility criteria. The second recruitment phase lasted seven months (Sept 2022 to Mar 2023), during which four GPs distributed 475 letters (121 of these were to previously contacted individuals). From this, 26 individuals contacted the Research Team (letter response rate: 5.5%) and 15 participants enrolled in the trial (letter enrolment rate: 3.2%), which equalled one participant enrolled per 32 letters distributed; this increased efficiency several-fold (ratio: 6.6) compared with the first recruitment phase. A further 28 individuals from secondary recruitment strategies, were screened of which nine enrolled in the trial. | |
| *First recruitment phase*   - GP recruitment letters – *n* = 4 - Community group recruitment – *n* = 2 | *Second recruitment phase*   - GP recruitment letters – *n* = 15 - Community group recruitment – *n* = 1 - Social media – *n* = 1 - Participant referrals – *n* = 3 - Internal University recruitment – *n* = 4 |
| *Narrative discussion of the challenges with recruitment*  Recruitment began in March 2022, after the Covid-19 pandemic had caused changes in routine GP health screening. Under normal circumstances, GPs are recommended to monitor fasting plasma glucose or HbA1c levels every six to twelve months in individuals at high risk for T2D (such as those with prediabetes) (NICE, 2017). Our initial recruitment strategy, therefore, aimed to capture individuals with prediabetes based upon recent health screening results. In the first six months of the pandemic, primary care health testing (which includes routine blood tests) fell by 80% (The Health Foundation, 2020). There was a further restriction on non-clinically urgent blood tests in 2021 (including prediabetes screening) (NHS England, 2021), which was imposed by NHS England due to a shortage in blood sample collection vials caused by pandemic-related supply chain issues. Collectively, this meant that prospective participants had been sent recruitment letters based upon their pre-pandemic prediabetes status, and when subsequently screened during visit one they no longer had a HbA1c value in the prediabetic range. | |

National Institute for Health and Care Excellence (NICE) (2017). Type 2 diabetes: prevention in people at high risk. Available from: <https://www.nice.org.uk/guidance/PH38/chapter/Recommendations#risk-identification-stage-1>

NHS England (2021). Becton Dickinson blood specimen collection portfolio supply disruption: Recommended actions for medical directors, nursing directors, GPs and pathology laboratories to optimise resources for pathology laboratory work [PAR888]. Dated 10, August 2021. Available from: [www.england.nhs.uk/wp-content/uploads/2021/08/B0888-becton-dickinson-blood-specimen-collection-portfolio-supply-disruption-v2.pdf](http://www.england.nhs.uk/wp-content/uploads/2021/08/B0888-becton-dickinson-blood-specimen-collection-portfolio-supply-disruption-v2.pdf)

The Health Foundation (2020). Use of primary care during the COVID-19 pandemic. Available from: <https://www.health.org.uk/news-and-comment/charts-and-infographics/use-of-primary-care-during-the-covid-19-pandemic>

| **Table S2.** Study outcomes and prioritisation. |
| --- |
| ***Feasibility and tolerability outcomes*** |
| *Primary outcome*   - Adherence to the intervention   *Secondary outcomes*   - Recruitment - Attrition rate - Side effects (GASE questionnaire) - Blinding to the intervention (-1, 0, +1 scale) |
| ***Exploratory outcomes*** |
| - Bodyweight (kg) - Body mass index (kg/m^2^) - Waist circumference (m) - Handgrip strength (kg) - Glycated haemoglobin (HbA_1c_) (mmol/mol and %) - Plasma glucose (mmol/L) - Serum insulin (pmol/L) - Serum C-peptide (pmol/L) - Homeostatic model assessment of insulin sensitivity (HOMA2–%S) - Homeostatic model assessment of *β*-cell function (HOMA2–%B) - Homeostatic model assessment of insulin resistance (HOMA2-IR) - Quantitative insulin sensitivity check index (QUICKI) - Plasma fructosamine (µmol/L) - Plasma high sensitivity C-reactive protein (hsCRP) (mg/L) - Serum lipids: total cholesterol, HDL, LDL, and triglycerides (mmol/L) - Serum lipid ratios: LDL:HDL and TC:HDL - Plasma methylglyoxal (MGO) (ng/mL) - Plasma 4-hydroxy-2-nonenal (HNE) (ng/mL) - Plasma, serum, and urine markers of kidney and liver function - Diastolic, systolic, and mean arterial blood pressures - Transthoracic echocardiographic outcomes:   - Resting heart rate (bpm)   - Cardiac output (L/min)   - Stroke volume index (mL/m^2^)   - Cardiac index (L/min/m^2^)   - Stroke volume (mL)   - Stroke volume index (mL/m^2^)   - Ejection fraction (%)   - Right and left ventricle dimensions (cm)   - End systolic and diastolic volumes (mL) - Serum apolipoprotein A1 and B* - N-terminal pro-brain natriuretic peptide (NT-proBNP)* - Estimated glomerular filtration rate (eGFR) (mL/min/1.73m^2^)* - Plasma and urine markers of carnosine and carnosinase metabolism* |
| HDL, high density lipoprotein; LDL, low density lipoprotein; TC, total cholesterol.  *These outcomes were not explored due to the absence of change in other markers and the change in study population. This included several TTE parameters, which are not reported above, but can be found in the study preregistration ([NCT05329610](https://classic.clinicaltrials.gov/ct2/show/NCT05329610)). |

| **Table S3.** Biochemistry assay coefficient of variation. | | | |
| --- | --- | --- | --- |
| **Biochemical marker** | | | |
| *Intra-assay repeatability* | CV% |  | CV% |
| Plasma glucose (mmol/L) | 0.8% | Serum creatinine (µmol/L) | 1.7% |
| Plasma fructosamine (µmol/L) | 0.9% | Plasma GGT (U/L) | 5.5% |
| Plasma hs-CRP (mg/L) | 1.1% | Plasma LDH (U/L) | 1.2% |
| Serum cholesterol (mmol/L) | 0.5% | Plasma albumin (g/L) | 0.7% |
| Serum LDL (mmol/L) | 3.2% | Plasma total bilirubin (µmol/L) | 3% |
| Serum HDL (mmol/L) | 0.7% | Plasma total protein (g/L) | 0.8% |
| Serum triglycerides (mmol/L) | 1.2% | Plasma urea (mmol/L) | 0.9% |
| Plasma ALP (U/L) | 0.7% | Urinary glucose (mmol/L) | 7.8% |
| Plasma ALT (U/L) | 1.1% | Urinary albumin (mg/L) | 12.1%* |
| Plasma AST (U/L) | 1.3% | Urinary creatinine (mmol/L) | 0.4%* |
| Plasma creatine kinase (U/L) | 0.7% | Urinary urea (mmol/L) | 0.6%* |
|  |  |  |  |
| *ELISA inter-assay repeatability* |  |  |  |
| Serum insulin (pmol/L) | 17.5% |  |  |
| Serum C-peptide (pmol/L) | 7.2% |  |  |
| ALP, alkaline phosphatase; ALT, alanine transaminase; AST, aspartate transaminase; CV%, coefficient of variation; ELISA, enzyme-linked immunosorbent assay; GGT, gamma-glutamyl transferase; HDL, high density lipoprotein; hsCRP, high sensitivity C-reactive protein; LDL, low density lipoprotein. *Values from 30 samples in duplicate; remaining CVs include all 54 samples measured in duplicate. | | | |

These values were generated internally from duplicate samples across all concentration ranges. In the event of a sample falling outside of acceptable coefficient of variation (CV%) limits (<10%), additional analyses were performed (*e.g.,* samples in triplicates or quadruplicates). Intra-assay CV% were within acceptable limits (<10%) for 22 of the 23 biochemistry outcomes (95.7%), with urinary albumin slightly outside of these limits (12.1%); inter-assay CV% was within acceptable limits (<15%) for one of the two ELISAs, with serum insulin slightly outside of these limits (Table S3). Due to insufficient reagents or test kits, internal CV% were not performed for HbA1c, MGO, or HNE. Instead, manufacturer-reported data show inter-assay CV% <3% for HbA1c at the lower range of T2D concentrations (HbA1c: 7%), and intra- and inter-assay CV% <10% for HNE and MGO (concentration range not specified).

| **Table S4.** Adherence for observed values and predictive distributions. | | |
| --- | --- | --- |
| **Timepoint** | **Placebo** | ***β*-alanine** |
| *1-month* |  |  |
| Observed values | 0.93 (0.87 to 0.96) | 0.93 (0.89 to 0.96) |
| Predicted values | 0.95 (0.69 to 1.0) | 0.95 (0.74 to 1.0) |
| *2-months* |  |  |
| Observed values | 0.91 (0.85 to 0.95) | 0.92 (0.86 to 0.95) |
| Predicted values | 0.94 (0.66 to 1.0) | 0.95 (0.67 to 1.0) |
| *Follow-up* |  |  |
| Observed values | 0.91 (0.84 to 0.95) | 0.92 (0.85 to 0.95) |
| Predicted values | 0.94 (0.66 to 1.0) | 0.94 (0.66 to 1.0) |
| Data presented as median (95%CrI). | | |

| **Table S5.** Predictive distributions for participant recruitment. | | | |  |
| --- | --- | --- | --- | --- |
| **Probability of achieving** | ***n* = 50** | ***n* = 75** | ***n* = 100** | |
| Randomisation (80%) | 200 | 295 | 390 | |
| Evaluation (80%) | 223 | 330 | 435 | |
| Randomisation (90%) | 220 | 320 | 420 | |
| Evaluation (90%) | 245 | 362 | 476 | |

| **Table S6.** Clinical biochemical outcomes with default priors. | | | | | | |
| --- | --- | --- | --- | --- | --- | --- |
| **Outcome** | **Placebo** | | ***β*-alanine** | | **Intervention Effect** | **Probability > 0** |
|  | **Baseline** | **Follow-Up** | **Baseline** | **Follow-Up** | **Placebo:*β*-alanine** |  |
| ALP (U/L) | 81 ± 28 | 81 ± 27 | 82 ± 30 | 82 ± 31 | 0.98 (-6.9 to 8.9) | 0.599 |
| ALT (U/L) | 33 ± 16 | 33 ± 14 | 29 ± 12 | 30 ± 18 | 0.46 (-7.8 to 8.9) | 0.548 |
| AST (U/L) | 32 ± 8 | 31 ± 5 | 30 ± 5 | 31 ± 10 | 0.33 (-5.4 to 6.0) | 0.547 |
| Creatine kinase (U/L) | 172 ± 91 | 169 ± 87 | 163 ± 79 | 155 ± 69 | -6.7 (-38.8 to 25.8) | 0.333 |
| Creatinine (µmol/L) | 97 ± 24 | 94 ± 22 | 92 ± 17 | 92 ± 19 | 3.0 (-1.5 to 7.2) | 0.910 |
| GGT (U/L) | 23 ± 16 | 29 ± 19 | 26 ± 17 | 28 ± 22 | -3.9 (-14.9 to 7.2) | 0.236 |
| LDH (U/L) | 323 ± 98 | 331 ± 105 | 295 ± 66 | 311 ± 40 | -3.0 (-49.0 to 44.1) | 0.446 |
| Albumin (g/L) | 42.4 ± 2.0 | 41.5 ± 1.9 | 43.6 ± 2.6 | 42.3 ± 2.4 | 0.05 (-1.3 to 1.4) | 0.687 |
| Total bilirubin (µmol/L) | 11.4 ± 4.3 | 11.7 ± 5.5 | 13.0 ± 6.0 | 13.1 ± 6.6 | -0.07 (-3.0 to 2.9) | 0.409 |
| Total protein (g/L) | 69.4 ± 2.9 | 69.2 ± 4.2 | 70.9 ± 5.2 | 70.3 ± 5.0 | -0.07 (-2.8 to 2.8) | 0.481 |
| Urea (mmol/L) | 5.1 ± 1.4 | 4.7 ± 1.1 | 4.2 ± 1.3 | 4.6 ± 1.3 | 0.48 (-0.24 to 1.2) | 0.903 |
| Urinary creatinine (mmol/L) | 11.5 ± 6.5 | 12.2 ± 6.3 | 9.0 ± 5.9 | 9.0 ± 6.8 | -1.3 (-4.8 to 2.2) | 0.228 |
| Urinary albumin (mg/L) | 49.2 ± 133.7 | 159.6 ± 525.8 | 21.7 ± 57.9 | 12.9 ± 31.2 | -54.5 (-169.4 to 59.2) | 0.168 |
| UACR (mmol/mol) | 3.3 ± 7.4 | 10.8 ± 34.1 | 2.0 ± 3.8 | 1.1 ± 2.4 | -5.0 (-13.1 to 3.2) | 0.110 |
| *Urinary albumin (mg/L) | 12.3 ± 14.4 | 13.9 ± 25.6 | 6.3 ± 5.6 | 4.7 ± 5.9 | -3.5 (-16.1 to 9.0) | 0.285 |
| *UACR (mmol/mol) | 1.3 ± 1.6 | 1.4 ± 2.4 | 1.0 ± 1.3 | 0.5 ± 0.6 | -0.71 (-1.9 to 0.47) | 0.116 |
| Urinary glucose (mmol/L) | 0.26 ± 0.13 | 0.34 ± 0.21 | 0.23 ± 0.17 | 0.23 ± 0.17 | -0.10 (-0.23 to 0.04) | 0.079 |
| Urinary urea (mmol/L) | 238 ± 112 | 242 ± 91 | 176 ± 96 | 192 ± 113 | -11.8 (-81.5 to 58.6) | 0.371 |
| Outcome data presented as mean ± 1 SD; Bayesian ANCOVA inferential data presented as median (95%CrI); the probability shows the proportion of the posterior distribution that is beyond zero (i.e., p = 0.5 means the posterior distribution contains an equal proportion above and below zero). ALP, alkaline phosphatase; ALT, alanine transaminase; AST, aspartate transaminase; GGT, gamma-glutamyl transferase; LDH, lactate dehydrogenase; urinary creatinine:albumin ratio (UACR). *Results with exclusion of one outlier from each group, see the corresponding text in the results section for details. | | | | | | |

| **Table S7.** CNAP and TTE cardiovascular outcomes. | | | | | | |
| --- | --- | --- | --- | --- | --- | --- |
| **Outcome** | **Placebo** | | ***β*-alanine** | | **Intervention Effect** | **Probability > 0** |
|  | **Baseline** | **Follow-Up** | **Baseline** | **Follow-Up** | **Placebo:*β*-alanine** |  |
| *CNAP outcomes* |  |  |  |  |  |  |
| Systolic BP (mmHg) | 138 ± 13 | 133 ± 20 | 132 ± 15 | 132 ± 16 | 2.2 (-11.4 to 15.9) | 0.629 |
| Diastolic BP (mmHg) | 80 ± 8 | 77 ± 13 | 75 ± 8 | 77 ± 10 | 2.7 (-6.8 to 12.2) | 0.721 |
| Mean Arterial BP (mmHg) | 102 ± 8 | 99 ± 14 | 99 ± 11 | 100 ± 11 | 2.8 (-7.1 to 12.8) | 0.717 |
| *TTE outcomes - structure* |  |  |  |  |  |  |
| IVSd (cm) | 1.0 ± 0.2 | 1.0 ± 0.1 | 0.9 ± 0.2 | 0.8 ± 0.2 | -0.10 (-0.24 to 0.05) | 0.101 |
| LVIDd (cm) | 5.0 ± 0.4 | 5.2 ± 0.6 | 4.9 ± 0.5 | 4.9 ± 0.6 | -0.17 (-0.58 to 0.25) | 0.211 |
| LVPWd (cm) | 1.0 ± 0.1 | 1.1 ± 0.2 | 1.0 ± 0.1 | 0.9 ± 0.2 | -0.08 (-0.22 to 0.05) | 0.107 |
| LVIDs (cm) | 3.4 ± 0.7 | 3.4 ± 0.6 | 3.4 ± 0.4 | 3.3 ± 0.5 | -0.12 (-0.49 to 0.27) | 0.264 |
| LVOT (cm) | 2.9 ± 0.3 | 2.9 ± 0.3 | 2.9 ± 0.2 | 2.9 ± 0.2 | -0.01 (-0.18 to 0.15) | 0.454 |
| RVOT (cm) | 3.1 ± 0.2 | 3.1 ± 0.3 | 3.0 ± 0.2 | 3.0 ± 0.3 | -0.08 (-0.30 to 0.14) | 0.231 |
| *TTE outcomes - function* |  |  |  |  |  |  |
| Resting heart rate (bpm) | 66 ± 14 | 62 ± 12 | 64 ± 11 | 60 ± 8 | -0.74 (-6.3 to 4.8) | 0.393 |
| Stroke volume (mL) | 66 ± 21 | 68 ± 21 | 70 ± 19 | 72 ± 14 | 0.88 (-9.1 to 10.3) | 0.573 |
| SVI (mL/m^2^) | 31 ± 8 | 32 ± 8 | 34 ± 9 | 35 ± 6 | 1.3 (-3.0 to 5.6) | 0.712 |
| Cardiac output (L/min) | 4.1± 1.0 | 4.1 ± 1.3 | 4.4 ± 1.0 | 4.2 ± 0.7 | -0.28 (-0.98 to 0.42) | 0.207 |
| Cardiac index (L/min/m^2^) | 2.0 ± 0.4 | 2.0 ± 0.5 | 2.2 ± 0.5 | 2.1 ± 0.4 | -0.07 (-0.40 to 0.24) | 0.324 |
| Ejection fraction (%) | 57 ± 7 | 58 ± 8 | 59 ± 4 | 58 ± 7 | -0.98 (-6.3 to 4.5) | 0.355 |
| ESV (mL) | 50 ± 17 | 51 ± 17 | 50 ± 13 | 53 ± 15 | 2.2 (-7.3 to 11.7) | 0.681 |
| ESV/BSA (mL/m^2^) | 24 ± 7 | 24 ± 6 | 24 ± 6 | 26 ± 7 | 1.6 (-3.3 to 6.4) | 0.745 |
| EDV (mL) | 116 ± 33 | 121 ± 32 | 120 ± 30 | 125 ± 25 | 1.4 (-13.0 to 16.0) | 0.577 |
| EDV/BSA (mL/m^2^) | 55 ± 12 | 57 ± 11 | 58 ± 13 | 61 ± 11 | 1.8 (-5.0 to 8.5) | 0.708 |
| Outcome data presented as mean ± 1 SD; Bayesian ANCOVA inferential data presented as median (95%CrI); the probability shows the proportion of the posterior distribution that is beyond zero (i.e., p = 0.5 means the posterior distribution contains an equal proportion above and below zero). BP; blood pressure, BSA, body surface area; EDV, end diastolic volume; EF, ejection fraction; ESV, end systolic volume; IVSd, interventricular septal end diastole; LVIDd, left ventricular internal diameter end diastole; LVIDs, left ventricular internal diameter end systole; LVOT, left ventricular outflow tract; LVPWd, left ventricular posterior wall end diastole; RVOT, right ventricular outflow tract; SVI, stroke volume index. | | | | | | |
